# Population structure of drug-resistant *Mycobacterium tuberculosis* in Central Asia

**DOI:** 10.1186/s12879-019-4480-7

**Published:** 2019-10-29

**Authors:** Anna Engström, Uladzimir Antonenka, Abdylat Kadyrov, Gulmira Kalmambetova, Katharina Kranzer, Matthias Merker, Olim Kabirov, Nargiza Parpieva, Asliddin Rajabov, Evgeni Sahalchyk, Zayniddin Sayfudtinov, Stefan Niemann, Harald Hoffmann

**Affiliations:** 1 Molecular and Experimental Mycobacteriology, Research Center Borstel, Parkallee 1, 23845 Borstel, Germany; 2 Department of Medical Biochemistry and Microbiology, Uppsala University, IMBIM, Box 582, 751 23 Uppsala, Uppsala Sweden; 3 Present address: Clinical Genomics, Science for Life Laboratory, Tomtebodavägen 23 A, 17165 Solna, Sweden; 4 Present address: School of Engineering Sciences in Chemistry, Biotechnology and Health, Division of Gene Technology, KTH Royal Institute of Technology, Stockholm, Sweden; 5 Postal address: Science for Life Laboratory, Tomtebodavägen 23 A, 17165 Solna, Sweden; 6 WHO Supranational Reference Laboratory of Tuberculosis, IML red GmbH, Institute of Microbiology and Laboratory Medicine, Robert Koch-Allee 2, D-82131 Gauting, Germany; 7 National Tuberculosis Institute, 90a Ahuunbaen Street, 720075 Bishkek, Kyrgyzstan; 8 National Reference Laboratory of Tuberculosis, 90a Ahuunbaen Street, 720075 Bishkek, Kyrgyzstan; 9 Clinical Research Department, London School of Hygiene and Tropical Medicine, London, UK; 10 National Reference Laboratory of Tuberculosis, Vakhdat District, Dushanbe, Tajikistan; 11 Republican Specialized Scientific and Practical Medical Center of Tuberculosis and Pulmonology, Alimov Str.1, Tashkent, Uzbekistan 100086; 12 National TB Center, National Tuberculosis Program, Dushanbe, Tajikistan; 13 National Reference Laboratory of Tuberculosis, Alimov Str.1, Tashkent, Uzbekistan 100086; 14 SYNLAB Gauting, SYNLAB Human Genetics Munich, Robert Koch-Allee 2, 82131 Gauting, Germany; 15 Kuratorium Tuberculosis in the World e.V, Robert Koch-Allee 2, 82131 Gauting, Germany

**Keywords:** Molecular typing, MIRU, MDR-TB, Beijing, Cluster

## Abstract

**Background:**

Drug-resistant tuberculosis (TB) is a major public health concern threathing the success of TB control efforts, and this is particularily problematic in Central Asia. Here, we present the first analysis of the population structure of *Mycobacterium tuberculosis* complex isolates in the Central Asian republics Uzbekistan, Tajikistan, and Kyrgyzstan.

**Methods:**

The study set consisted of 607 isolates with 235 from Uzbekistan, 206 from Tajikistan, and 166 from Kyrgyzstan. 24-loci MIRU-VNTR (Mycobacterial Interspersed Repetitive Units - Variable Number of Tandem Repeats) typing and spoligotyping were combined for genotyping. In addition, phenotypic drug suceptibility was performed.

**Results:**

The population structure mainly comprises strains of the Beijing lineage (411/607). 349 of the 411 Beijing isolates formed clusters, compared to only 33 of the 196 isolates from other clades. Beijing 94–32 (*n* = 145) and 100–32 (*n* = 70) formed the largest clusters. Beijing isolates were more frequently multidrug-resistant, pre-extensively resistant (pre-XDR)- or XDR-TB than other genotypes.

**Conclusions:**

Beijing clusters 94–32 and 100–32 are the dominant MTB genotypes in Central Asia. The relative size of 100–32 compared to previous studies in Kazakhstan and its unequal geographic distribution support the hypothesis of its more recent emergence in Central Asia. The data also demonstrate that clonal spread of resistant TB strains, particularly of the Beijing lineage, is a root of the so far uncontroled MDR-TB epidemic in Central Asia.

## Background

Drug-resistant tuberculosis (TB) is a public health concern threathing the success of TB control efforts. This is particularily problematic in the Central Asian Republics (CARs) Uzbekistan, Tajikistan, and Kyrgyzstan where multidrug-resistant (MDR)-TB rates reach 24%, 22%, 27%, and 63%, 45% and 60% among new and previously treated cases, respectively [1].

Little is known about the population structure of drug resistant *Myocbacterium tuberculosis* (MTB) strains in Central Asia. Available reports are limited by either outdated genotyping techniques, sampling of special populations and/or small sample sizes [2–4]. Here, we provide a snapshot of the population structure of MTB strains based on World Health Organization resistance surveys performed in Kyrgyzstan, Tajikistan, and Uzbekistan representing two thirds of the Central Asian population.

For genotyping we combined 24-loci MIRU-VNTR typing (Mycobacterial Interspersed Repetitive Units - Variable Number of Tandem Repeats) with spoligotyping. MIRU-VNTR provides high-resolution discrimination of strains for epidemiological studies and phylogenetic classification [5]. MIRU-VNTR data allow also to generate parsimonious phylogenetic networks such as minimum-spanning trees (MST) [6]. These results were combined with phenotypic drug susceptibility data to determine the presence of highly resistant dominant clones.

## Methods

Seven hundred and seven clinical MTB isolates (306 from Uzbekistan, 216 from Tajikistan, and 185 from Kyrgyzstan) were collected in the framework of cross-sectional national drug resistance surveys (DRS) [7, 8] and included in the study. The study population was biased towards resistant strains since only isolates that required re-testing for quality control purposes as per the WHO DRS protocol which was valid at the time were included [8]. This approach artificially increased the proportion of MDR-,pre-extensively drug-resistant (pre-XDR) and XDR-TB strains. Ethical approvals for the DRSs were granted by the respective national ethical committees.

DNA was extracted by heat lysis of bacteria from solid culture on LJ medium. 24-loci MIRU-VNTR genotyping and spoligotyping was performed as previously described [5, 9]. Genotypes were identified on the MIRU-VNTR*plus* web database (www.miruvntrplus.org) [5]. Spoligotyping data were used to confirm strain relationships and for genotype classification. Typing data was analysed with Bionumerics (v7.5; Applied Maths, Sint-Martens-Latem, Belgium). MST analysis based on MIRU-VNTR data was performed using the categorical coefficient. For each 24-loci MIRU-VNTR pattern a unique multiple loci VNTR analysis (MLVA) MtbC15–9 haplotype was assigned by using the MIRU-VNTR*plus* nomenclature [10]. A cluster was defined as a minimum of two isolates harboring identical genotyping patterns from different patients. Patients with mixed molecular typing patterns were excluded. Drug susceptibility testing was performed by the resazurin microtiter assay [11].

## Results

Twenty four-loci MIRU-VNTR typing and spoligotyping were successfully performed for 607 isolates, while 76 isolates (62 from Uzbekistan, seven from Tajikistan, and seven from Kyrgyzstan) yielded indeterminate results. For 23 isolates (9 from Uzbekistan, three from Tajikistan, and 11 from Kyrgyzstan), mixed genotyping patterns were observed. One isolate from Kyrgyzstan had incomplete phenotypic data.

The final study set consisted of 607 isolates of which 235 (38.7%) originated from Uzbekistan, 206 (33.9%) from Tajikistan, and 166 (27.3%) from Kyrgyzstan (Table 1). The male to female ratio of patients was 1.5 and ages ranged from 12 to 85 years with a mean of 37.9 years (SD ±15.7). Across the three countries, 333 (54.9%) were new and 221 (36.4%) previously treated cases (for 53 patients information regarding previous treatments was missing; Table 1). Thirty five isolates (5.8%) were pan-susceptible, 293 (48.3%) MDR-, pre-XDR, or XDR-TB, while the remaining 279 isolates (46.0%) had any other resistance pattern. MDR- and XDR-TB were more prominent among Kyrgyz (33%) and Tajik (35%) than among Uzbek isolates (20%), which was mainly attributable to different re-testing algorithms in the DRS protocols.

**Table 1:** Demographic data, genotypes, haplotypes and phenotypes of the Central Asian *M. tuberculosis* complex study population

	All countries			Uzbekistan			Tajikistan			Kyrgystan		
	No. strains	%	SD	No. strains	%	SD	No. strains	%	SD	No. strains	%	SD
*Samples*	607			235	38.7		206	33.9		166	27.3	
*Gender*												
Male		359	59.1	139	59.1		120	58.3		100	60.2	
Female		239	39.4	96	40.9		85	41.3		58	34.9	
Unknown		9		0			1			8		
*Age* ^*a*^												
Mean	37.94		15.67	42.91		16.6	34.53		14.36	34.18		13.49
*Treatment*												
New	333	54.9		156	66.4		93	45.1		84	50.6	
Previous	221	36.4		79	33.6		113	54.9		29	17.5	
Unknown	53			0			0			53		
*MIRU lineage*												
Beijing	411	67.7		136	57.9		154	74.8		121	72.9	
Dehli/CAS	6	1		6	2.6							
H37Rv-like	60	9.9		31	13.2		12	5.8		17	10.2	
LAM	36	5.9		22	9.4		5	2.4		9	5.4	
NEW-1	31	5.1		16	6.8		10	4.9		5	3	
URAL	28	4.6		10	4.3		11	5.3		7	4.2	
Haarlem	26	4.3		7	3		13	6.3		6	3.6	
S-type	3	0.5		2	0.9					1	0.6	
TUR	1	0.2					1	0.5				
X-type	1	0.2		1	0.4							
Undefined	4	0.7		4	1.7							
*MLVA MtbC15–9*												
94–32 (Beijing)	145	23.9		58	24.7		49	23.8		38	22.9	
100–32 (Beijing)	70	11.5		12	5.1		51	24.8		7	4.2	
*Resistance pattern*												
Pan-susceptible	35	5.8		21	8.9		2	1		12	7.2	
Other resistances	279	46		127	54		66	32		86	51.8	
MDR-TB	172	28.3		47	20		71	34.5		54	32.5	
Pre XDR-TB	94	15.5		34	14.5		50	24.3		10	6	
XDR-TB	27	4.4		6	2.6		17	8.3		4	2.4	

^a^Unkown for Tajikistan and 23 unkown for Kyrgystan

*SD* standard deviation

**Fig. 1:**
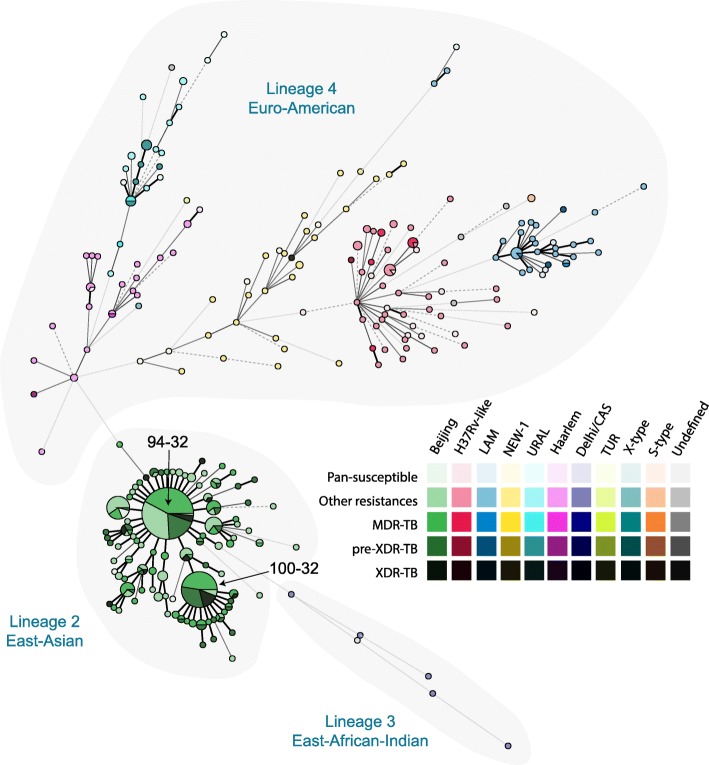
Minimum spanning tree based on the 24-loci MIRU-VNTR typing data of 607 MTB isolates from Uzbekistan, Tajikistan and Kyrgyzstan. The size of each circle is proportional to the number of MIRU-VNTR types belonging to a particular complex. Classification of the isolates into the different phylogenetic lineages and resistance patterns is visualized by color coding

Based on the MIRU-VNTR profiles and spoligotyping patterns, 603 isolates were classified into previously described lineages (Table 1). 411 (67.7%) isolates belonging to lineage 2 (East-Asian; Beijing genotype) [12]. Six (1.0%) isolates belonged to lineage 3 (East-African-Indian; Delhi/CAS genotype), and 186 (30.6%) isolates belonged to lineage 4 (Euro-American) with the predominiating genotypes H37Rv-like (60; 9.9%), LAM (36; 5.9%), NEW-1 (31; 5.1%), URAL (28; 4.6%), and Haarlem (26; 4.3%). Four (0.7%) isolates could not be linked to a previously described lineage and were classified as “undefined”. An MST was calculated (Fig. 1) confirming the UPGMA (unweighted pair group method with arithmetic mean) tree-based genotype classification of MIRU-VNTR*plus* (data not shown).

Cluster analysis revealed that 382 (62.9%) of the 607 isolates shared a genotyping pattern with at least one other isolate. They were grouped in 38 clusters ranging from two to 145 isolates. The two largest clusters had haplotypes 94–32 (*n* = 145; 23.9%) and 100–32 (*n* = 70; 11.5%) (Fig. 1). The prevalence of 94–32 was equally distributed over the CARs whereas cluster 100–32 was particularly prominent in Tajikistan (51 strains; 72.9%). 349 (84.9%) of the 411 Beijing isolates formed clusters, compared to only 33 (16.8%) of the 196 isolates from other clades.

**Fig. 2:**
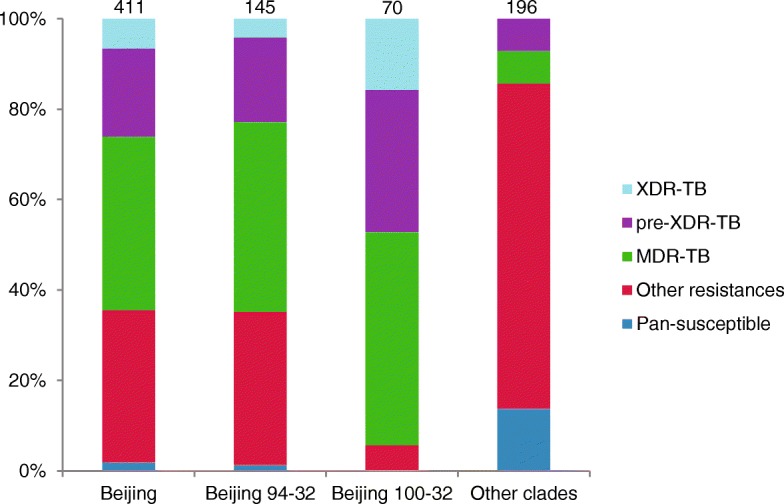
Proportions of isolates based on drug resistance patterns. The number of isolates with a given genotype/MLVA MtbC15–9 haplotype is given above the corresponding column

Beijing strains were more frequently MDR-, pre-XDR- or XDR-TB (265/411; 64.5%) than strains of other genotypes (28/196; 14.3%; no XDR-TB) (Fig. 2). Beijing cluster 94–32 showed the same drug resistance pattern as other Beijing strains, whereas cluster 100–32 was almost completely (66/70; 94.3%) MDR, pre-XDR, or XDR.

## Discussion

High prevalence of Beijing strains has previously been reported from Central Asia [4]. The star-like shape of the lineage 2/Beijing population in Fig. 1 with two central clones surrounded by layers of single and multi-locus variants is a typical pattern for emerging and expanding MTB populations [13]. The two largest clusters 94–32 and 100–32 were found in all three Central Asian countries and represented more than a third of all study isolates indicating strong population expansion across borders. Our findings confirm the results of a global Beijing population study which reported that 94–32 and 100–32 were predominantly prominent in Uzbekistan, Russia and Eastern European countries and had particularly high clustering rates [13]. The smaller size of 100–32 and its unequal distribution to the three CARs suggest however its more recent arrival in Central Asia and a shorter regional expansion time than of 94–32. This hypothesis is further supported by Mokrousov [14] and Skibe et al. [15] who have already reported 100–32 as minor group (< 4%) of Beijing isolates in Central Asia while it reached proportions of over 10% and over 20% in Eastern Europe and Russia, respectively, and while Skibe et al. observed 94–32 as dominant cluster in Kazakhstan. The extent of cross-border spread and locally restricted transmisison networks can however not be ultimately resolved with MIRU-VNTR cluster data and require further investigation with higher resolution.

High percentages of MDR among Beijing strains has also been reported from other Eastern European countries, like Georgia, Kazakhstan, and Uzbekistan [4, 15, 16]. Higher proportions of MDR- and XDR-TB were also observed within the 100–32 genotype by Merker and colleagues [13]. The authors analyzed the full genomes of MDR-TB strains belonging to MLVA-VNTR haplotypes 94–32 and 100–32 and showed that the maximal divergences were 17 and 23 SNPs, respectively, within genomes of those clusters [13].

Our data indicate that spread of resistant MTB strains, particularly of lineage 2/Beijing, is an important root of the ongoing MDR-TB epidemic in Central Asia. MIRU-VNTR is a powerful tool for investigating population structures and transmission dynamics, however, advances in the field of whole genome sequencing have resulted in molecular methods that can give better resolution and should ideally be used to elucidate specific transmission pathways. Analyzing the full genomes of this or an unbiased and thus even more representative study population could provide an even deeper insight into the MTB population structure in CAR.

## Conclusions

Clusters 94–32 and 100–32 are the dominant genotypes driving population expansion of Beijing strains in the CARs Uzbekistan, Tajikistan, and Kyrgyzstan. The data demonstrate that spread of resistant TB strains, particularly of the Beijing lineage and its clusters 94–32 and 100–32, is an important root of the ongoing MDR-TB epidemic in Central Asia. Measures to combat this epidemic need to be improved.

## Data Availability

The datasets used and/or analysed during the current study are available from the corresponding author on reasonable request.

## Abbreviations

CAR: Central Asian Republic
DRS: Drug resistance surveys
MDR: Multi-drug-resistant / resistance
MIRU-VNTR: Mycobacterial Interspersed Repetitive Units - Variable Number of Tandem Repeats
MLVA: Multiple loci VNTR analysis
MST: Minimum-spanning tree
MTB: *Myocbacterium tuberculosis*
TB: Tuberculosis
UPGMA: Unweighted pair group method with arithmetic mean
XDR: Extensively drug resistant / extensive drug resistance
